# Phosphatidylethanolamine-binding protein is not involved in μ-opioid receptor-mediated regulation of extracellular signal-regulated kinase

**DOI:** 10.3892/mmr.2015.3161

**Published:** 2015-01-08

**Authors:** JIA-MING BIAN, NING WU, RUI-BIN SU, JIN LI

**Affiliations:** 1 State Key Laboratory of Toxicology and Medical Countermeasures, Beijing Key Laboratory of Neuropsychopharmacology, Beijing Institute of Pharmacology and Toxicology, Beijing 100850, P.R. China; 2 Department of Pharmacology, Military General Hospital of Beijing People’s Liberation Army, Beijing 100700, P.R. China

**Keywords:** phosphatidylethanolamine-binding protein, μ-opioid receptor, extracellular signal-regulated kinase, G protein-coupled receptors

## Abstract

Stimulation of the μ-opioid receptor activates extracellular signal-regulated kinase (ERK), however, the mechanism by which this occurs remains to be elucidated. Phosphatidylethanolamine-binding protein (PEBP) has been reported to act as a negative regulator of the ERK cascade (Raf-MEK-ERK) by binding to Raf-1 kinase. In the present study, the role of PEBP in μ-opioid receptor-mediated ERK activation was investigated in Chinese hamster ovary/μ cells and SH-SY5Y cells, as well as in human embryonic kidney 293 cells expressing other types of G protein-coupled receptors. The acute activation of μ-opioid receptors by morphine or (D-Ala^2^, MePhe^4^, Gly^5^-ol) enkephalin induced a rapid activation of ERK. Prolonged morphine treatment did not affect the phosphorylation level of ERK compared with control cells, but the phosphorylation level of ERK decreased markedly when cells were precipitated with naloxone following chronic morphine treatment. For the phosphorylation of PEBP, no change was identified under the designated drug treatment and exposure duration. A total of two other types of G protein-coupled receptors, including Gs-coupled dopamine D1 receptors and Gq-coupled adrenergic α1A receptors were also investigated and only the activation of adrenergic α1A receptors induced an upregulated phosphorylation of PEBP, which was protein kinase C activity dependent. Thus, PEBP did not have a significant role in μ-opioid receptor-mediated regulation of ERK.

## Introduction

The μ-opioid receptor, a member of the G protein-coupled receptor (GPCR) family characterized by a seven-transmembrane structure, has been extensively investigated (1). Activation of μ-opioid receptors leads to the inhibition of adenylyl cyclase (AC) and thus the cyclic adenosine monophosphate (cAMP)/protein kinase A (PKA) pathway is suppressed (2). The prolonged opioid exposure results in a comprehensive adaptation of opioid receptor trafficking and signaling, including receptor downregulation and desensitization (3). Furthermore, naloxone precipitation-induced cAMP overshoot following chronic opioid treatment has become an approved marker of cellular opioid dependence (4). In addition to inhibiting the cAMP/PKA pathway through the Gαi/o subunit, the μ-opioid receptor was also revealed to crosstalk with the extracellular signal-regulated kinase (ERK) cascade (Raf-MEK-ERK). The stimulation of opioid receptors in cells may induce a notable enhancement of phosphorylated ERK (pERK), which results in the activation of the ERK cascade (5,6). Furthermore, ERK activation was observed in the neurons in several reward-associated brain regions and the administration of the MEK inhibitor eliminated long-term potentiation in the hippocampus, which is considered a classic example of synaptic plasticity (7).

The crosstalk between the μ-opioid receptor pathway and the ERK cascade involves multiple important molecules, including PKA (8), β-arrestins (9) and protein kinase C (PKC) (10), although the exact mechanism remains to be elucidated. PKC is a potent activator of the ERK cascade. Early studies suggested that PKC directly phosphorylated and activated Raf-1 (11,12), however, subsequent studies indicated that PKC activated the ERK cascade through a Ras-dependent pathway rather than direct phosphorylation of Raf-1 (13,14).

Phosphatidylethanolamine-binding protein (PEBP), also termed Raf kinase inhibitor protein, was identified to be an endogenous negative regulator of the ERK cascade by binding to Raf-1 kinase but not the B-Raf isoform (15,16). The phosphorylation of PEBP at Ser153 induced by PKC resulted in the release of PEBP from Raf-1 kinase and thus the PEBP-induced inhibition of the ERK cascade was rescued (17). Previously, our research group found that hippocampal PEBP was involved in morphine dependence in rats and downregulation of hippocampal PEBP levels induced by antisense oligodeoxynucleotides resulted in aggravated morphine dependence (18). Due to the crucial role of ERK in drug dependence, it is possible that PEBP phosphorylation serves as a mechanism involved in μ-opioid receptor-mediated ERK activation.

## Materials and methods

### Cell lines

The present study was approved by the ethics committee of Beijing Institute of Pharmacology and Toxicology (Beijing, China). SH-SY5Y cells were obtained from the Cell Culture Center, Chinese Academy of Medical Science (Beijing, China) and maintained in Dulbecco’s modified Eagle’s medium (DMEM)/F12 medium (Gibco-BRL, Carlsbad, CA, USA) supplemented with 10% fetal bovine serum (Gibco-BRL), 100 U/ml penicillin and 100 U/ml streptomycin (Sigma-Aldrich, St. Louis, MO, USA) at 37°C in a humidified atmosphere containing 5% CO_2_. Chinese hamster ovary (CHO) cells, which stably express the rat μ-opioid receptor (CHO/μ cells) were setup previously (19) and were maintained in the same conditions as that for SH-SY5Y cells with the addition of 200 μg/ml geneticin. Human embryonic kidney (HEK)293 cells that stably express the human D1 dopamine receptor or α1A adrenergic receptor were setup as previously described (20,21) and cultured in DMEM medium, supplemented with the same as that for CHO/μ cells.

### Drug administration

All the drugs used in the present study, including morphine, D-Ala^2^, MePhe^4^, Gly^5^-ol enkephalin (DAMGO), naloxone, Gö6983, dopamine and phenylephrine (PE) were purchased from Sigma-Aldrich. For acute drug treatment, cells were seeded onto six-well plates for 24 h at densities of 5×10^5^ cells/well (CHO/μ cells and HEK 293 cells) or 1×10^6^ cells/well (SH-SY5Y cells), and were serum-starved overnight upon reaching 80% confluence prior to stimulation by different concentration of drugs or vehicles. For morphine chronic treatment, cells were seeded onto six-well plates for 12 h prior to treatment, at densities of 2×10^5^ cells/well (CHO/μ cells) or 5×10^5^ cells/well (SH-SY5Y cells), and then treated with morphine or vehicle for 36 h.

### Immunoblotting analysis

Immunoblot was performed following drug treatment as described previously (22). Briefly, following stimulation, cells were washed twice with chilled phosphate-buffered saline (Sigma-Aldrich). The cells were placed on ice and 60 μl chilled lysis buffer [50 mM Tris, 150 mM NaCl, 1 mM EDTA, 1 mM EGTA, 1% NP-40, 1 mg/l aprotinin, 1 mg/l pepstatin, 1 mg/l leupeptin, 1 mM phenylmethylsulfonyl fluoride, 1 mM dithiothreitol, 2 mM NaF and 1 mM sodium vanadate (pH 7.4; Sigma-Aldrich)] per well was added. Cells were scraped from plates and transferred to a 1.5-ml Eppendorf tube. Following incubation on ice for 30 min, the lysis was centrifuged at 14,000 × g for 20 min at 4°C. Supernatant protein concentrations were determined using a Bicinchoninic Acid Protein Assay kit (Pierce Biotechnology, Inc., Rockford, IL, USA). Aliquots of sample were boiled for 5 min in the presence of 1X loading buffer (Pierce Biotechnology, Inc.). 40 μg of protein was resolved using SDS-PAGE on 15% tricine gels and then was transferred onto a polyvinylidene difluoride membrane (Millipore, Billerica, MA, USA) for immunoblotting. Rabbit monoclonal antibody against PEBP (1:1,000) and rabbit monoclonal antibody against phospho-PEBP (S153) (1:200) were obtained from Abcam (Cambridge, UK). Mouse monoclonal antibody against phospho-ERK1/2 (1:2,000) and rabbit polyclonal antibody against ERK1/2 (1:5,000) were obtained from Santa Cruz Biotechnology, Inc. (Santa Cruz, CA, USA). Subsequently, the phosphorylated proteins were visualized and the phospho-antibodies were stripped from the blots by incubating in stripping buffer for 1 h at 37°C. Blots were subsequently reblocked and probed with antibodies against ERK or PEBP. For ERK or PEBP activity, the quantity of pERK (combined pERK1 and 2) or pPEBP protein was normalized to total ERK (combined ERK1 and 2) or PEBP.

### Statistical analysis

Statistical analysis was performed using GraphPad Prism^®^ version 5.02 (GraphPad Software Inc., La Jolla, CA, USA). All data are expressed as the mean ± standard error of the mean and optical density values were determined using a gel imaging system (Alpha Innotech, San Leandro, CA, USA). Student’s t-test was used to compare the differences between two groups and statistics between groups were assessed using analysis of variance followed by Dunnett’s-test. P<0.05 was considered to indicate a statistically significant difference.

## Results

### Acute opioid treatment induces transient ERK, but not PEBP phosphorylation in CHO/μ cells and SH-SY5Y cells

**Figure 1:**
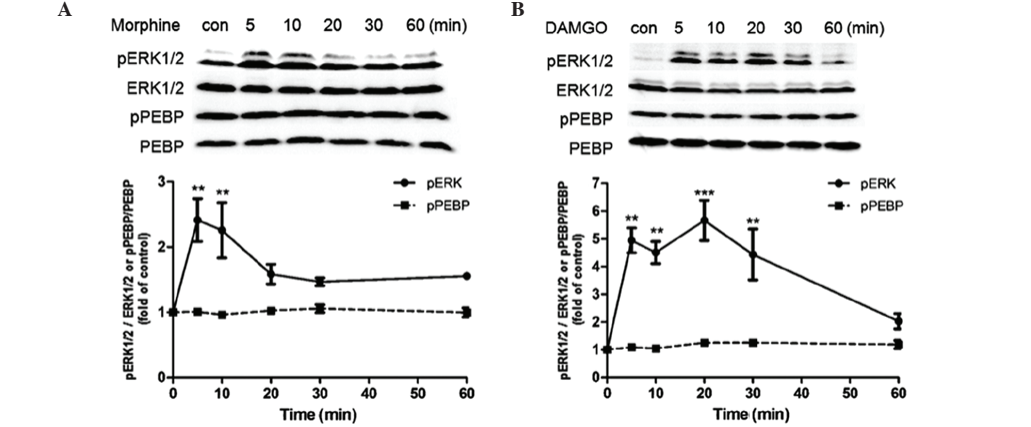
Effect of opioid treatment on ERK and PEBP phosphorylation in CHO/μ cells. (A) Cells were treated with 1 μM morphine for the indicated time intervals. (B) Cells were treated with 1 μM DAMGO for the indicated time intervals. Data are presented as the mean ± standard error of the mean from three independent experiments. One-way analysis of variance was used followed by Dunnett’s post hoc test. ^**^P<0.01 and ^***^P<0.001, compared with the con. PEBP, phosphatidylethanolamine-binding protein; DAMGO, (D-Ala^2^, MePhe^4^, Gly^5^-ol) enkephalin; ERK, extracellular signal-regulated kinase; CHO cells, Chinese hamster ovary cells; con, control.

Initially, a CHO cell line was selected that expressed exogenous rat μ-opioid receptors (2.9 pmol/mg membrane protein) to examine the effect of acute opioid exposure on the phosphorylation of ERK and PEBP. It was observed that 1 μM morphine caused transient activation of ERK at 5 min (~2-fold compared with the control treatment), which then decreased to the basal level. However, no significant difference in expression of pPEBP was observed during 1 h morphine treatment (Fig. 1A). Similarly to morphine, 1 μM DAMGO also induced rapid ERK activation, which was sustained for a longer time period than that induced by morphine (>30 min); however, no significant difference in pPEBP was observed during 1 h of DAMGO treatment (Fig. 1B). To confirm that the activation of ERK is mediated by the μ-opioid receptor, the μ-opioid receptor antagonist Naloxone (5 μM) was selected to preincubate cells for 30 min prior to DAMGO treatment and the result revealed that naloxone completely eradicated DAMGO-induced ERK activation (data not shown), indicating that the activation of ERK was mediated by μ-opioid receptors.

**Figure 2:**
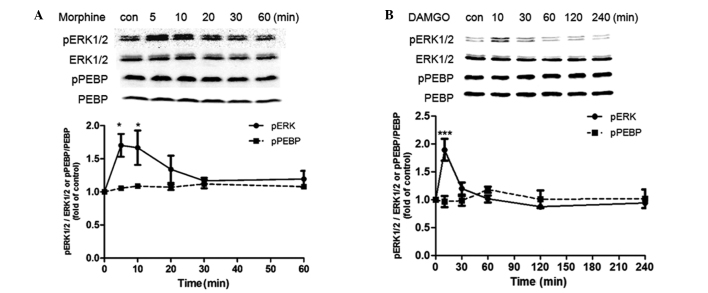
Effect of opioid treatment on ERK and PEBP phosphorylation in SH-SY5Y cells. (A) Cells were treated with 1 μM morphine for the indicated time intervals. (B) Cells were exposed to 1 μM DAMGO for the indicated time intervals. Data are presented as the mean ± standard error of the mean from three independent experiments. One-way analysis of variance was used followed by Dunnett’s post hoc test. ^*^P<0.05 and ^***^P<0.001, compared with the con. PEBP, phosphatidylethanolamine-binding protein; DAMGO, (D-Ala^2^, MePhe^4^, Gly^5^-ol) enkephalin; ERK, extracellular signal-regulated kinase; CHO cells, Chinese hamster ovary cells; con, control.

A similar investigation was also conducted in SH-SY5Y cells. Acute treatment with 10 μM morphine for 1 h caused transient activation of ERK at 5 min (~1.7-fold compared with the control treatment), which returned to basal levels after 30 min. During this time course, however, the level of pPEBP exhibited little change compared with the control treatment (Fig. 2A). For DAMGO treatment, a 4 h treatment duration was investigated. It was found that DAMGO induced a transient activation of ERK at 10 min (~1.9-fold compared with the control treatment), which then returned to basal levels after 30 min, but the level of pPEBP did not alter significantly during 4 h of DAMGO treatment (Fig. 2B).

### Chronic morphine treatment has no effect on PEBP expression and phosphorylation

**Figure 3:**
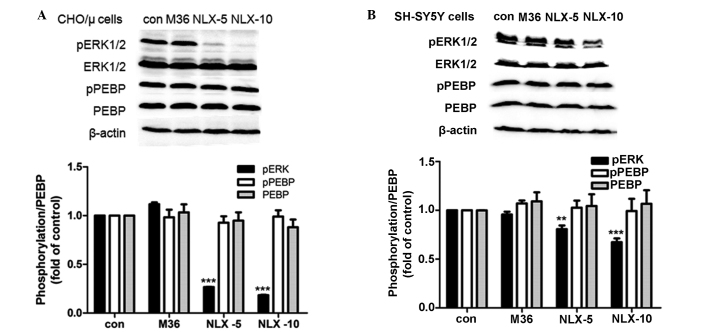
Effect of chronic morphine treatment on the phosphorylation of ERK and PEBP and the expression of PEBP in (A) CHO/μ cells and (B) SH-SY5Y cells. Cells were treated with 10 μM morphine (M36) or vehicle (Con) for 36 h and were then washed twice with culture medium and the withdrawal was preceded by precipitating the cells with 5 μM naloxone for 5 min (NLX-5) or 10 min (NLX-10). Data are presented as the mean ± standard error of the mean from three independent experiments. One-way analysis of variance was used followed by Dunnett’s post hoc test. ^**^P<0.01 and ^***^P<0.001, compared with the con. PEBP, phosphatidylethanolamine-binding protein; DAMGO, (D-Ala^2^, MePhe^4^, Gly^5^-ol) enkephalin; ERK, extracellular signal-regulated kinase; CHO cells, Chinese hamster ovary cells; con, control.

Since short-term opioid treatment did not induce any significant change in pPEBP, whether chronic treatment with morphine may affect the level of PEBP or pPEBP in these two cell lines was investigated. It was found that chronic treatment with 10 μM morphine for 36 h did not affect PEBP expression level in CHO/μ cells and SH-SY5Y cells, as well as the level of pPEBP. When the cells were precipitated with 5 μM naloxone following chronic morphine treatment, significantly decreased phosphorylation of ERK was observed after 10 or 20 min of naloxone precipitation and furthermore, the decreased phosphorylation of ERK in CHO/μ cells (Fig. 3A) was greater than that in SH-SY5Y cells (Fig. 3B). However, the level of pPEBP did not alter in the two cell lines during the course of naloxone precipitation (Fig. 3A and B).

### Opioid-induced ERK phosphorylation is inhibited by Gö6983

**Figure 4:**
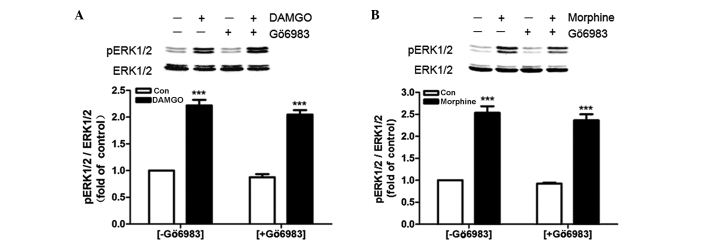
Effect of Gö6983 on opioid-induced ERK activation in SH-SY5Y cells. Cells were pretreated with 1 μM Gö6983 or vehicle (dimethyl sulfoxide) for 30 min and were then stimulated with (A) 1 μM DAMGO or (B) 1 μM morphine for 5 min. Data are presented as the mean ± standard error of the mean from three independent experiments. Two-way analysis of variance was used followed by the Bonferroni post hoc test. ^***^P<0.001, compared with each control. PEBP, phosphatidylethanolamine-binding protein; DAMGO, (D-Ala^2^, MePhe^4^, Gly^5^-ol) enkephalin; ERK, extracellular signal-regulated kinase; CHO cells, Chinese hamster ovary cells.

Pretreatment with Gö6983 (1μM) for 30 min did not eradicate the rapid activation of ERK induced by 1 μM DAMGO (Fig. 4A) or 1 μM morphine (Fig. 4B).

### Activation of the adrenergic α1A receptor but not the dopamine D1 receptor induces phosphorylation of PEBP

**Figure 5:**
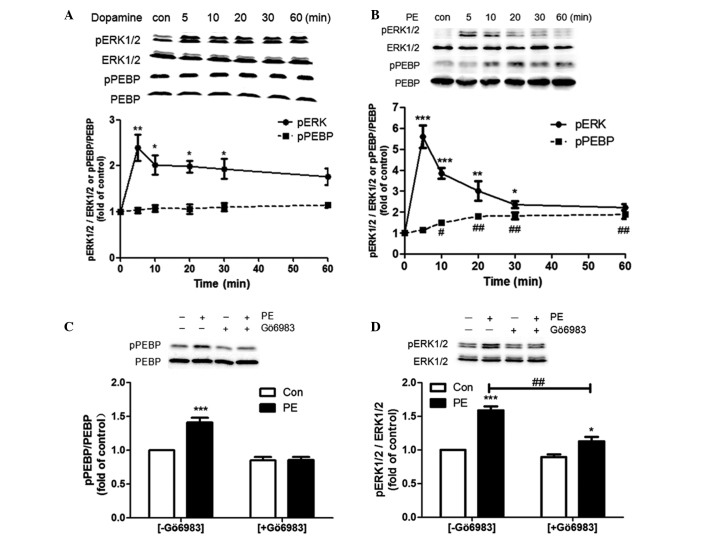
Effect of the activation of the dopamine D1 receptor and adrenergic α1A receptor on ERK and PEBP phosphorylation in HEK293 cells. (A) HEK293/D1 cells were treated with 1 μM dopamine for the indicated time intervals. (B) HEK293/α1A cells were treated with 1 μM PE for the indicated time intervals. One-way analysis of variance was used followed by Dunnett’s post hoc test. For pERK, ^*^P<0.05, ^**^P<0.01 and ^***^P<0.001, compared with the Con; for pPEBP, ^#^P<0.05 and ^##^P<0.01, compared with the Con. (C and D) HEK293/α1A cells were pretreated with 1 μM Gö6983 or vehicle (dimethyl sulfoxide) for 30 min and were then stimulated with 1 μM PE for 30 min. Two-way analysis of variance was used followed by the Bonferroni post hoc test. ^*^P<0.05 and ^***^P<0.001, compared with each Con. Student’s t-test was used for the ERK activity comparison with or without Gö6983 (^##^P<0.01). All data are presented as the mean ± standard error of the mean from three independent experiments. PEBP, phosphatidylethanolamine-binding protein; PE, phenylephrine; ERK, extracellular signal-regulated kinase; CHO cells, Chinese hamster ovary cells; HEK, human embryonic kidney; Con, control.

The present study continued to investigate whether the activation of two other types of GPCR may affect the phosphorylation of PEBP. A total of two HEK293 cell lines that stably express dopamine D1 receptors (HEK293/D1 cells, 2.9 pmol/mg membrane protein) and adrenergic α1A receptors (HEK293/α1A cells, 0.6 pmol/mg membrane protein) were used. For D1 dopamine receptors, 1 μM dopamine induced rapid and sustained ERK phosphorylation during 60 min treatment with a peak at 5 min but did not induce any change in pPEBP compared with the control treatment (Fig. 5A). For the α1A adrenergic receptor, a Gq-coupled receptor, rapid activation of ERK and also a significant upregulation of pPEBP was observed during 60 min of treatment with 1 μM PE (Fig. 5B). The ERK activation was rapid with a peak (5–6 fold of control) at 5 min and then a significant decrease (~2 fold of control) until 30 min. The phosphorylation of PEBP gradually increased following the phosphorylation of ERK. Gö6983 preincubation completely eradicated PE-induced PEBP phosphorylation (Fig. 5C) and significantly reduced the level of ERK phosphorylation (Fig. 5D).

## Discussion

The crosstalk between opioid receptors and ERK has undergone comprehensive investigation, however, the underlying mechanism remains to be elucidated. PEBP is a small (21–23 kDa) soluble protein, which is widely expressed in cells and tissues (23) and was previously identified to be a negative regulator of ERK activation (15). However, the role of PEBP in μ-opioid receptor-mediated activation of ERK remains to be elucidated. PKC activity appears to be involved in opioid-induced ERK activation, due to specific evidence, which suggested that DAMGO induced PKC activation in SH-SY5Y cells by stimulating μ-opioid receptors (24) and (D-Pen2, D-Pen5)-enkephalin induced PKC activation in NG-108 cells by stimulating δ opioid receptors (25). Since PKC-induced phosphorylation of PEBP results in the disinhibition of Raf-1 signaling, which leads to the activation of ERK (26), it is possible that μ-opioid receptor-mediated activation of PKC may induce the phosphorylation of PEBP, which then regulates the activity of ERK through Raf-1 kinase. However, the present study demonstrated that activation of the μ-opioid receptor did not regulate the phosphorylation of PEBP.

In the present study, two cell lines that express different levels of μ-opioid receptors were used. CHO/μ cells were selected as they express high levels of μ-opioid receptors, which is an advantage for the functional study of opioids, while SH-SY5Y cells were selected due to their similarity to the neurons that express endogenous μ-opioid receptors (27). Morphine and DAMGO are two well-investigated agonists of the μ-opioid receptor, characterized by differing structure and efficacy. The intracellular signaling mechanisms mediated by DAMGO are often largely different from that mediated by morphine, including receptor phosphorylation, internalization and desensitization (28–31), therefore, these two agonists were used to activate μ-opioid receptors in the present study.

The data presented in the present study provide evidence that morphine and DAMGO induce a rapid activation of ERK; however, no significant phosphorylation of PEBP was observed following short-term opioid treatment in CHO/μ cells and SH-SY5Y cells. Kramer *et al* (24) reported a significant PKC translocation to the cell membrane of SH-SY5Y cells after 4 h of DAMGO stimulation (~2-fold of control), therefore, 4 h was selected as the end point of the observation. However, a corresponding elevation of pPEBP was not observed and the phosphorylation of ERK induced by DAMGO was only augmented in the first 30 min and then returned to the basal level until the end of stimulation. The present findings suggested that μ-opioid receptor-mediated rapid ERK activation was not associated with PEBP phosphorylation and short-term stimulation of μ-opioid receptor did not induce the change in pPEBP levels, even with different agonists.

Since PKC activation caused the activation of ERK, whether μ-opioid receptor-induced rapid ERK activation involved PKC activity was assessed in the present study. Gö6983 is a selective inhibitor for the majority of PKC isoenzymes, including PKC α, β, γ, δ and ξ (32), among which PKC α, β, γ and ξ were responsible for PEBP phosphorylation at Ser153 (17). Therefore, the application of Gö6983 may inhibit PKC-induced PEBP phosphorylation. It was found that DAMGO and morphine-induced ERK activation in SH-SY5Y cells was independent of Gö6983-sensitive PKC activity. A similar result was also obtained in rat cortical astrocytes in a study by Belcheva *et al* (33).

Long-term opioid treatment may cause a comprehensive adaption of opioid receptor trafficking and signaling, including AC superactivation. It has been reported that sustained morphine treatment augmented forskolin-stimulated cAMP formation (34,35) and the withdrawal using naloxone following chronic opioid treatment led to cAMP overshoot (36). The present study found that prolonged morphine treatment had no effect on the phosphorylation level of ERK compared with that induced by the vehicle. However, a significant downregulation of pERK was observed in CHO/μ cells and SH-SY5Y cells that were precipitated with naloxone after 36 h of morphine treatment, which was also demonstrated in previous studies (37,38). *In vivo*, chronic morphine administration resulted in differential regulation of ERK activity in different brain regions, including a decrease in pERK in the cerebral cortex (39) and an increase in pERK at the spinal level (40). For a cell line, the acute elevation of intracellular cAMP resulting from naloxone precipitation may enhance PKA activity, acting as a negative regulator of Raf-1 kinase (10), possibly contributing to decreased phosphorylation of ERK. After 36 h of morphine treatment, it was observed that chronic morphine exposure had no effect on the expression level of PEBP, as well as the phosphorylation of PEBP. However, there are studies suggesting that PKC activity was upregulated *in vivo* following chronic morphine administration (41,42) and in addition no significant phosphorylation of PEBP was identified even in the cells precipitated with naloxone. The present results indicated that PEBP possibly did not contribute to the cellular adaptation induced by chronic morphine treatment through the alteration in either phosphorylation or expression, particularly in the modulation of ERK.

The possible modulation of PEBP phosphorylation by other types of GPCR evoked our interest. Besides Gi/o-coupled μ-opioid receptor, the Gs-coupled dopamine D1 receptor and Gq-coupled adrenergic α1A receptor were also investigated to examine the effect of receptor activation on PEBP phosphorylation. It was found that the activation of the dopamine D1 receptor induced sustained ERK activation, but failed to alter the level of pPEBP during 60 min of dopamine treatment, indicating that PEBP phosphorylation was not involved in the activation of ERK induced by the Gs-coupled receptor. Lefkowitz *et al* (43) has described a mechanism of Gs-dependent ERK activation: The activation of G_s_-coupled receptor induces accumulation of cAMP and Rap-1 is activated by cAMP and B-Raf is also activated by Rap-1, thus ERK is activated.

For the Gq-coupled adrenergic α1A receptor, however, activation leads to the elevation of intracellular diacylglycerol and Ca^2+^ (44), which are activators of PKC, therefore, it was expected that PE induced the phosphorylation of PEBP. It was found that PE-induced PEBP phosphorylation was delayed compared with ERK activation, similar to that induced by PMA in SH-SY5Y cells (data not shown), indicating that the rapid activation of ERK mediated by the adrenergic α1A receptor was not as a result of PEBP phosphorylation. However, it was unknown whether successive ERK phosphorylation following the acute phase was associated with PEBP phosphorylation. Inhibition of PKC completely eradicated PE-induced PEBP phosphorylation and significantly reduced the level of ERK activation, indicating that adrenergic α1A receptor-mediated PEBP and ERK phosphorylation are PKC activity dependent.

Taken together, the present results demonstrated that activation of the μ-opioid receptor does not modulate the phosphorylation of PEBP and PEBP did not contribute to GPCR-mediated rapid activation of ERK. Thus, PEBP may have a minor role in μ-opioid receptor-mediated ERK regulation.
